# Targeting BRD2 and BRD4 inhibit the growth of KSHV-infected immortalized endothelial cells through suppression of LANA translation

**DOI:** 10.1371/journal.ppat.1014288

**Published:** 2026-06-01

**Authors:** Jungang Chen, Jiaojiao Fan, Margaret Qin, Zhen Lin, Shengyu Mu, Lu Dai, Zhiqiang Qin

**Affiliations:** 1 Department of Pathology, Winthrop P. Rockefeller Cancer Institute, University of Arkansas for Medical Sciences, Little Rock, Arkansas, United States of America; 2 Little Rock Central High School, Little Rock, Arkansas, United States of America; 3 Department of Pathology, Tulane University Health Sciences Center, Tulane Cancer Center, New Orleans, Louisiana, United States of America; 4 Department of Pharmacology & Toxicology, University of Arkansas for Medical Sciences, Little Rock, Arkansas, United States of America; Vanderbilt University Medical Center, UNITED STATES OF AMERICA

## Abstract

Kaposi’s sarcoma-associated herpesvirus (KSHV) is the etiologic agent of several human cancers, including Kaposi’s sarcoma (KS) and primary effusion lymphoma (PEL), both of which still lack effective treatment options. Members of the bromodomain and extra-terminal domain (BET) family, especially bromodomain-containing protein 4 (BRD4), play important roles in RNA polymerase II–mediated transcriptional regulation and are required for the expression of many tumor-driving oncogenes in various cancer cells. Therefore, BET proteins have become attractive targets for anticancer drug development. Previous studies have demonstrated the high sensitivity of PEL cells to BET inhibitors, and BRD4 silencing effectively blocks tumor cell proliferation. In contrast, KSHV-infected immortalized endothelial cells display strong resistance to BET inhibitors, including (+)-JQ1. To further develop BRD-targeted therapies for KSHV-infected immortalized endothelial cells, we identified MZ-1 and SIM-1, two BRD4 PROTAC degraders, as effective inhibitors of cell growth in these cells. Mechanistically, these inhibitory effects depend on suppression of LANA translation through increased eIF2α phosphorylation in KSHV-infected cells. Similar LANA suppression was also observed following RNAi-mediated silencing of BRD2 or BRD4. Proteomic analysis identified unique protein candidates altered in MZ-1- and SIM-1-treated KSHV-infected immortalized endothelial cells compared with (+)-JQ1-treated cells. In summary, our study develops an effective strategy against KSHV-infected immortalized endothelial cells using selective BRD PROTACs, which may help improve therapeutic outcomes for KSHV-related malignancies in the future.

## Author summary

Proteolysis-targeting chimeras (PROTACs) are heterobifunctional small molecules composed of a protein-of-interest ligand, a linker, and an E3 ligase ligand. PROTAC molecules recruit an E3 ligase to the target protein, inducing ubiquitination and subsequent degradation through the proteasome pathway. Here, we report that two BRD4 PROTAC molecules, MZ-1 and SIM-1, effectively inhibit the growth of KSHV-infected immortalized endothelial cells, which are resistant to conventional BET/BRD4 inhibitors. Interestingly, we found that MZ-1 and SIM-1 reduce the expression of LANA, the major KSHV-encoded latent protein, in these cells. Further experiments demonstrated that this repression of LANA occurs not through transcriptional inhibition, protein degradation, or autophagy, but through increased eIF2α phosphorylation and suppression of LANA translation. In addition, proteomic analysis identified a subset of unique protein candidates altered by MZ-1 and SIM-1, but not by the BRD4 inhibitor (+)-JQ1, in KSHV-infected immortalized endothelial cells. Future studies will investigate the roles of these unique protein candidates in MZ-1/SIM-1–induced growth inhibition, which may reveal new therapeutic targets against KSHV-related malignancies, especially KS.

## Introduction

Kaposi’s sarcoma-associated herpesvirus (KSHV), also known as human herpesvirus 8 (HHV-8), is a principal causative agent of several cancers that arise in immunocompromised patients, including Kaposi’s sarcoma (KS) and primary effusion lymphoma (PEL) [1,2]. KS consists of spindle cells (tumor cells of endothelial origin), proliferating abnormal and leaky blood vessels, and extravasated red blood cells with hemosiderin deposits [1]. Clinically, KS lesions are classified into patch, plaque, nodule, and tumor stages, and individual patients often present with multiple lesion types. AIDS-associated KS (AIDS-KS) can manifest as an aggressive, disseminated disease affecting the skin, lymph nodes, and visceral organs. Despite the reduced incidence of KS in the era of combined antiretroviral therapy (cART) for HIV infection, KS remains the most common AIDS-associated malignancy and a leading cause of morbidity and mortality in this population [3,4]. Another KSHV-associated malignancy, PEL, consists of transformed B cells harboring KSHV and typically arises within the pleural or peritoneal cavities of immunosuppressed patients [2]. PEL is a rapidly progressing malignancy with a median survival time of approximately 6 months, even with combination chemotherapy [5]. Therefore, KSHV-induced malignancies remain a significant clinical challenge due to the lack of effective therapies.

Like other herpesviruses, KSHV establishes lifelong infection in host cells. It exhibits two phases: a latent phase, during which only a limited number of viral genes are expressed, and a lytic phase, during which most viral genes are expressed, leading to the production of infectious virions [1]. In more than 90% of KSHV-infected host cells, the virus persists in the latent phase. The latency-associated nuclear antigen (LANA), encoded by KSHV ORF73, is essential for viral genome replication, persistence, transcriptional regulation, and stable segregation of the viral episome to daughter cells during mitosis [6–8]. LANA is expressed in all KSHV-infected tumor cells. In addition to its essential role in latency, LANA functions as an oncoprotein and contributes to KSHV pathogenesis [7–9]. For example, LANA promotes proliferation of infected cells by acting as a transcriptional regulator and interferes with both innate and adaptive immune responses, facilitating immune evasion [10]. Given the central role of LANA in KSHV persistence, multiple efforts have been made to develop small-molecule inhibitors targeting LANA [11–13] or to repurpose existing drugs that inhibit LANA or LANA-associated cellular proteins [14,15]. However, these approaches have shown limited efficacy in suppressing LANA function or tumor cell growth.

Bromodomain-containing protein 4 (BRD4) is a key member of the bromodomain and extra-terminal domain (BET) family, which also includes BRD2, BRD3, and the testis-specific protein BRDT. These proteins play important roles in RNA polymerase II–mediated transcriptional regulation [16]. BRD4 is often required for the expression of c-Myc and other tumor-driving oncogenes in various cancer types, making it an attractive target for anticancer drug development. Previous studies have demonstrated that PEL cells are highly sensitive to BET inhibitors, and that BRD4 silencing effectively suppresses tumor cell proliferation and cell-cycle progression through inhibition of c-Myc [17]. Subsequent studies identified (+)-JQ1, a BRD4 inhibitor, as an epigenetic activator of KSHV lytic reactivation. This effect may be associated with disruption of Rad21-dependent conformational control of latency [18] and interference with Myc super-enhancer function [19]. Notably, the C-terminal domain (CTD) of LANA binds to mitotic chromosomes and interacts with chromatin-associated proteins, including BET family members such as BRD2 and BRD4 [20–22]. BRD4 has been shown to contribute to LANA recruitment to chromatin and to its transcriptional activity [21,22], while BRD2 may regulate LANA function as a transcriptional activator and can phosphorylate the LANA CTD [20]. In contrast to findings in PEL cells, our previous study demonstrated that KSHV-infected immortalized endothelial cells, such as TIVE-LTC, exhibit strong resistance to BET inhibitors, including (+)-JQ1 [23]. To further develop BRD-targeted therapies for KSHV-infected immortalized endothelial cells (a model of KS) and to elucidate the underlying mechanisms, we identified MZ-1 and SIM-1, two BRD4 PROTAC degraders, as effective inhibitors of cell growth in these cells. Mechanistically, these inhibitory effects are primarily mediated through suppression of LANA translation via increased eIF2α phosphorylation in KSHV-infected cells. Similar suppression of LANA was observed following RNAi-mediated silencing of BRD2 or BRD4. Furthermore, proteomic analysis comparing protein expression profiles among MZ-1-, SIM-1-, and (+)-JQ1-treated KSHV-infected immortalized endothelial cells identified a subset of unique protein candidates specifically altered by MZ-1 and SIM-1.

## Results

### Selective BET degraders but not BET inhibitors suppress the growth of KSHV-infected immortalized endothelial cells

Our previous study showed that BET inhibitors, including (+)-JQ1, effectively inhibited the growth of PEL cells (CC_50_ ~ 0.05 µM) but not KSHV-infected immortalized endothelial cells such as TIVE-LTC [23]. Here, we further examined and compared the potency of different BET inhibitors and degraders in inhibiting the growth of KSHV-infected immortalized endothelial cell lines, TIVE-LTC and iTIME.219. As shown in Table 1 and S1 Fig, both TIVE-LTC and iTIME.219 exhibited high resistance to all BET inhibitors tested, including (+)-JQ1, OTX-15, CPI-203, and MX-417 (CC_50_ > 50 µM). In contrast, two BET degraders, MZ-1 and SIM-1 — both BRD4-targeting PROTAC molecules — effectively inhibited the growth of TIVE-LTC and iTIME.219 cells (CC_50_ ~ 0.1–1.0 µM). However, other BRD4 PROTACs tested, including ARV-825 and dBET1, showed no inhibitory effects (Table 1). Notably, both MZ-1 and SIM-1 exhibited much weaker inhibitory effects on KSHV-negative TIME cells (CC_50_ > 200 µM).

**Table 1:** The potency of selective BET inhibitors or degraders in growth inhibition of KSHV-infected immortalized endothelial cells.

Cell lines	BET inhibitors CC_50_ (µM) ^a^				BET degraders CC_50_ (µM)			
	(+)-JQ1	OTX-15	CPI-203	MX-417	ARV-825	dBET1	MZ-1	SIM-1
**TIVE-LTC**	>50	>50	>50	>50	>50	>50	1.08	0.097
**iTIME.219**	~ 50	>50	>50	>50	~ 25	>50	0.74	0.025
**TIME**	- ^b^	–	–	–	–	–	>200	>200

^a^The CC_50_ represents the 50% cytotoxic concentration which was determined by the WST-1 assays.

^b^“-”: not determined.

### MZ-1 and SIM-1 treatments effectively reduce LANA expression in KSHV-infected immortalized endothelial cells

**Fig 1:**
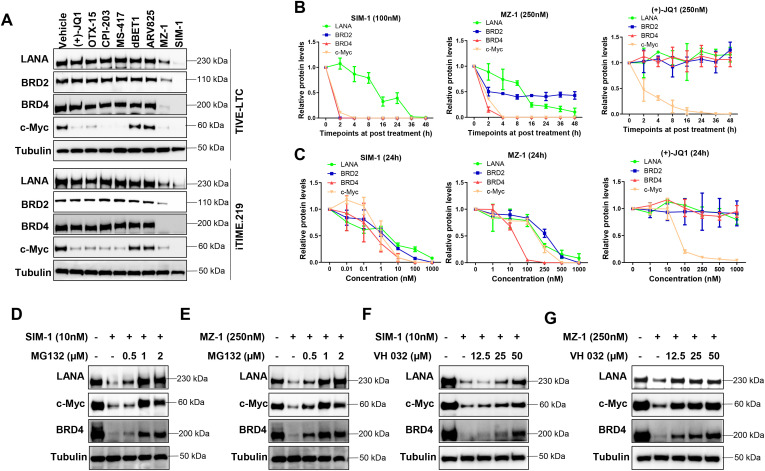
The impacts of selective BET inhibitors or degraders on target protein expression in KSHV-infected immortalized endothelial cells. **(A)** Cells were treated by different BET inhibitors or degraders at 100 nM for 24 h, then protein expression was measured by using Western blot. **(B-C)** TIVE-LTC were treated by indicated concentrations and time of SIM-1, MZ-1 or (+)-JQ1, respectively, then protein expression was measured by using Western blot and target protein levels were quantified by densitometry and normalized to the corresponding density of Tubulin protein using Image J. Error bars represent S.D. for 3 independent experiments. **(D-G)** TIVE-LTC were treated by SIM-1 or MZ-1 with or without indicated concentrations of MG132 or VH 032, respectively, then protein expression was measured by using Western blot.

To investigate why only MZ-1 and SIM-1 exhibited inhibitory effects, we examined the impact of these BET inhibitors and degraders on target protein expression in KSHV-infected immortalized endothelial cells by Western blot. Our results showed that only MZ-1 and SIM-1 markedly reduced BRD2 and BRD4 expression in TIVE-LTC and iTIME.219 cells, respectively, although most compounds tested reduced c-Myc expression (a major downstream effector of BRD4) (Fig 1A). Furthermore, only MZ-1 and SIM-1 effectively reduced LANA expression in both TIVE-LTC and iTIME.219 cells. Interestingly, both compounds also reduced LANA expression in *de novo* KSHV-infected HUVECs (S2 Fig). Time-course and dose-dependent experiments confirmed that MZ-1 and SIM-1 reduced the expression of LANA, BRD2, BRD4, and c-Myc, whereas (+)-JQ1 reduced only c-Myc expression in TIVE-LTC cells (Figs 1B, 1C and S3). Pretreatment with MG132, a proteasome inhibitor, restored BRD4, c-Myc, and LANA expression in MZ-1- or SIM-1-treated cells in a dose-dependent manner (Fig 1D and 1E). Similarly, pretreatment with VH032, a VHL ligand, also restored these protein levels (Fig 1F and 1G), consistent with the VHL-based PROTAC mechanism of MZ-1 and SIM-1. In contrast, MG132 or VH032 alone had no effect on LANA, BRD4, or c-Myc expression (S4 Fig).

### Direct knockdown of BRD2 or BRD4 proteins reduce LANA expression in KSHV-infected immortalized endothelial cells

**Fig 2:**
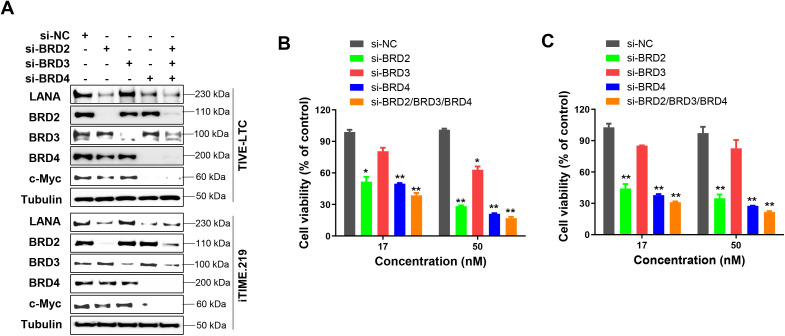
The impacts of direct knockdown of BET proteins on LANA expression in KSHV-infected immortalized endothelial cells. **(A)** Cells were transfected with BRD2-siRNA, BRD3-siRNA, BRD4-siRNA or non-target control siRNA (si-NC) for 72 h, then protein expression was measured by using Western blot. **(B-C)** Cell proliferation was measured by using the WST-1 assay. Error bars represent S.D. for 3 independent experiments, * = p < 0.05, ** = p < 0.01 (*vs* the si-NC control).

To determine which BET family members regulate LANA expression, we silenced BRD2, BRD3, or BRD4 using RNAi. Knockdown of either BRD2 or BRD4 reduced LANA expression, whereas BRD3 knockdown had no effect (Fig 2A). Only BRD4 knockdown reduced c-Myc expression, while knockdown of c-Myc did not affect LANA or BRD4 expression (S5 Fig), despite previous reports of interaction between LANA and c-Myc [24]. Pretreatment with MG132 or chloroquine (an autophagy inhibitor) did not restore LANA expression in BRD2- or BRD4-knockdown cells (S6 Fig), indicating that LANA repression is not mediated through proteasomal degradation or autophagy.

Additionally, RNAi-mediated knockdown of BRD2 or BRD4 (but not BRD3) significantly inhibited the growth of KSHV-infected immortalized endothelial cells (Fig 2B and 2C), with combined knockdown producing maximal inhibition. These findings indicate that BRD2 and BRD4 are key regulators of LANA expression in these cells.

### BRD2 and BRD4 depletion reduces LANA expression independently of transcriptional regulation

**Fig 3:**
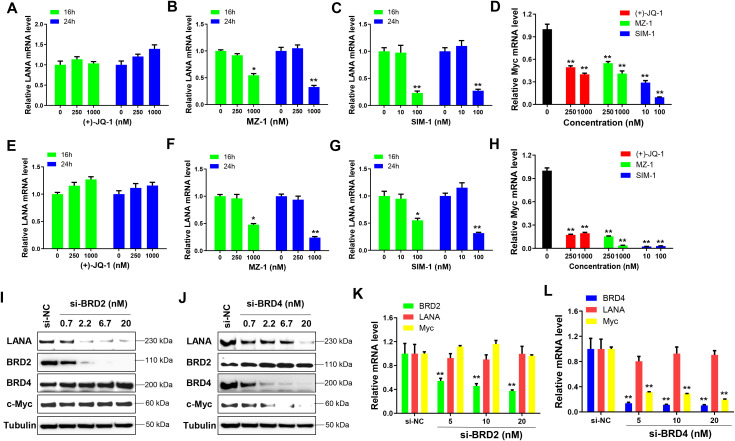
The impacts of selective BET inhibitors, degraders or BRDs siRNAs on LANA transcription in KSHV-infected immortalized endothelial cells. **(A-H)** TIVE-LTC **(A-D)** or iTIME.219 **(E-H)** were treated with indicated concentrations of SIM-1, MZ-1 or (+)-JQ1, respectively, then gene transcription was measured by using RT-qPCR. **(I-J)** TIVE-LTC were transfected with BRD2-siRNA, BRD4-siRNA or non-target control siRNA (si-NC) for 72 h, then protein expression was measured by using Western blot. **(K-L)** Gene transcription was measured by using RT-qPCR. Error bars represent S.D. for 3 independent experiments, * = p < 0.05, ** = p < 0.01 (*vs* the vehicle or si-NC control).

We next examined whether BRD2 or BRD4 depletion affects LANA transcription. RT-qPCR analysis showed that treatment with MZ-1, SIM-1, or (+)-JQ1 did not significantly alter LANA transcription (Fig 3A–3C and 3E–3G). Only very high concentrations of MZ-1 or SIM-1 reduced LANA transcription, likely due to cytotoxic effects. In contrast, all three compounds significantly reduced c-Myc transcription (Fig 3D and 3H). Similarly, although RNAi-mediated knockdown of BRD2 or BRD4 reduced LANA protein expression in a dose-dependent manner (Fig 3I and 3J), it did not affect LANA transcription (Fig 3K and 3L). These results indicate that BRD2/BRD4 depletion represses LANA expression at a post-transcriptional level.

### eIF2α phosphorylation mediates LANA repression induced by BRD2/BRD4 depletion

**Fig 4:**
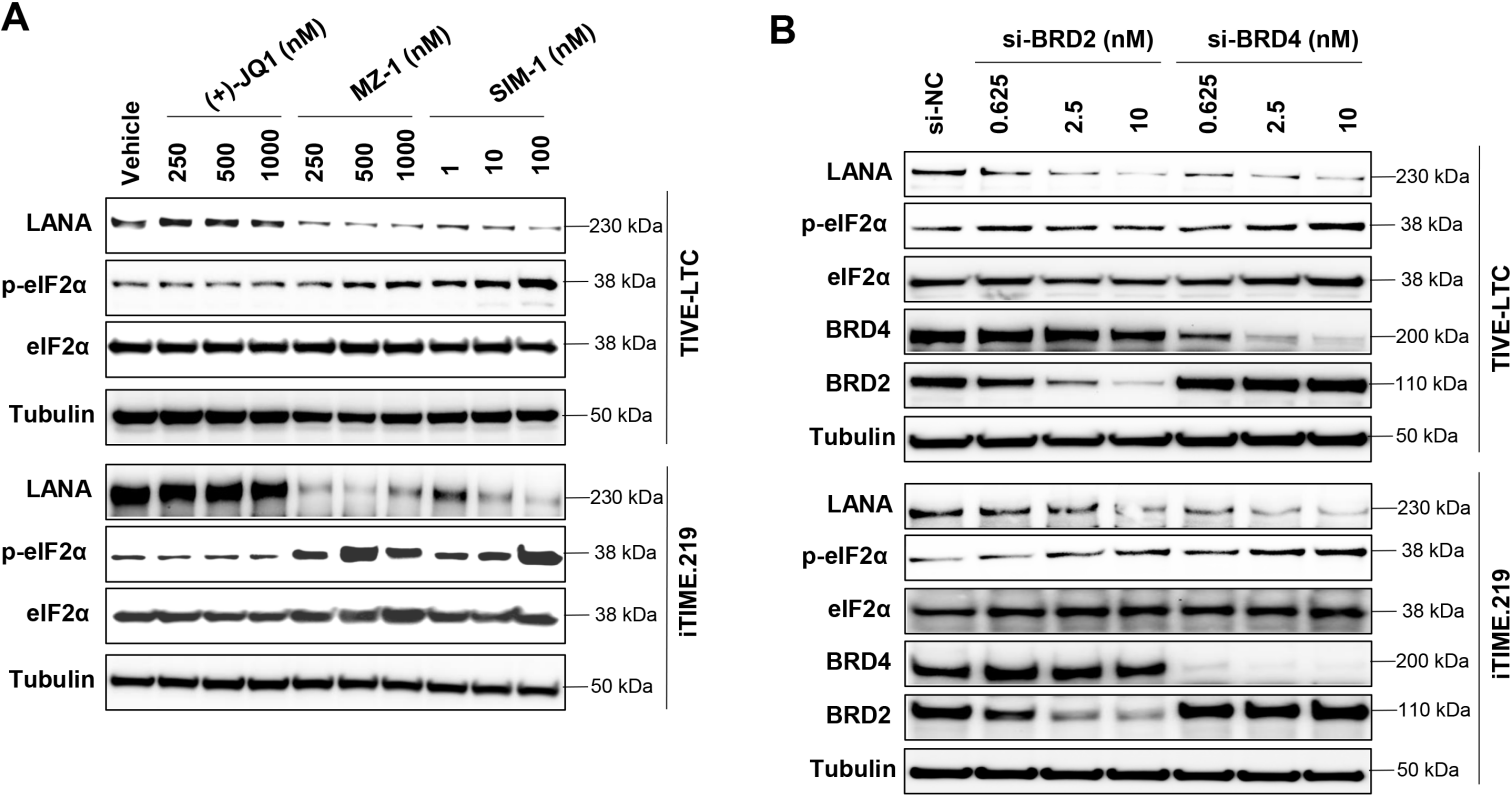
BRD2 or BRD4 depletion by PROTACs or RNAi increase the phosphorylation of eIF2α in KSHV-infected immortalized endothelial cells. **(A)** Cells were treated by indicated concentrations and time of SIM-1, MZ-1 or (+)-JQ1, then protein expression was measured by using Western blot. **(B)** Cells were transfected with BRD2-siRNA, BRD4-siRNA or non-target control siRNA (si-NC) for 72 h, then protein expression was measured by using Western blot.

We next investigated whether autophagy contributed to LANA repression. Pretreatment with autophagy inhibitors (bafilomycin A1 or chloroquine) did not restore LANA expression in MZ-1- or SIM-1-treated cells (S7 Fig), suggesting that repression occurs at the level of protein translation. eIF2α is a key regulator of translation initiation and a central component of the integrated stress response (ISR). Phosphorylation of eIF2α reduces global protein synthesis while allowing selective translation of stress-response genes [25,26]. We found that MZ-1 and SIM-1 significantly increased eIF2α phosphorylation in a dose-dependent manner in KSHV-infected immortalized endothelial cells, whereas (+)-JQ1 had no effect (Fig 4A). In contrast, only minimal increases were observed in KSHV-negative TIME cells (S8 Fig). Similarly, RNAi-mediated knockdown of BRD2 or BRD4 increased eIF2α phosphorylation (Fig 4B).

**Fig 5:**
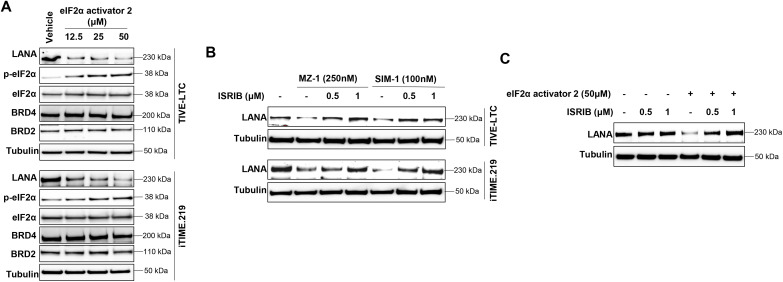
Activation of eIF2α is responsible for LANA repression in KSHV-infected immortalized endothelial cells. **(A)** Cells were treated by indicated concentrations of eIF2α activator 2, then protein expression was measured by using Western blot. **(B)** Cells were treated by SIM-1 or MZ-1 with or without ISRIB (an eIF2α inhibitor), then protein expression was measured by using Western blot. **(C)** TIVE-LTC were treated by ISRIB with or without eIF2α activator 2, then protein expression was measured by using Western blot.

To further confirm the role of eIF2α phosphorylation in LANA repression, we found that eIF2α activator 2 treatment was able to increase eIF2α phosphorylation and reduce LANA expression in KSHV-infected immortalized endothelial cells (Fig 5A). Conversely, ISRIB, an inhibitor of eIF2α signaling, restored LANA expression in cells treated with MZ-1, SIM-1, or the eIF2α activator (Fig 5B and 5C).

**Fig 6:**
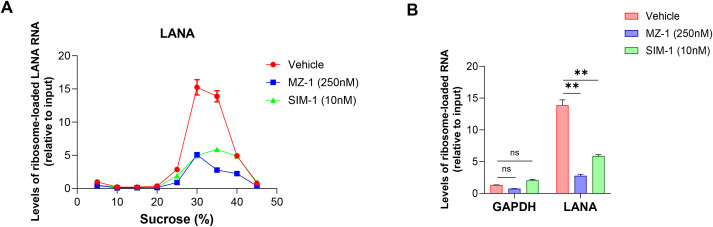
MZ-1 or SIM-1 treatments greatly reduce ribosome-loaded LANA mRNA in KSHV-infected immortalized endothelial cells. The iTIME.219 cells were treated with indicated concentrations of MZ-1 or SIM-1 for 24 h, then the polysome profiling assay was performed as described in the Methods. **(A)** RNAs from different ribosomal pellet layers were extracted using TRIzol and the levels of LANA mRNA were detected by using RT-qPCR. The levels of ribosome-loaded mRNA were normalized to the input. **(B)** The relative levels of LANA mRNA in the 35% polysome fraction were presented in parallel with GAPDH mRNA. Error bars represent S.D. for 2 independent experiments, ** = p < 0.01 (*vs* the vehicle control).

Because eIF2α phosphorylation is known to alter ribosome loading and the distribution of selective mRNAs [27], here we used polysome profiling assay to determine whether MZ-1 or SIM-1 can affect the binding of LANA mRNA with polysome. Our results showed that MZ-1 or SIM-1 treatment greatly reduced the ribosome-loaded of LANA mRNA when compared to the vehicle control (Fig 6A), further demonstrating that MZ-1 and SIM-1 inhibited the translation efficiency of LANA. As a comparison, MZ-1 or SIM-1 treatment could not affect the ribosome-loaded of GAPDH mRNA (Fig 6B).

### Global protein profile changed by selective BET inhibitors or degraders in KSHV-infected immortalized endothelial cells

**Fig 7:**
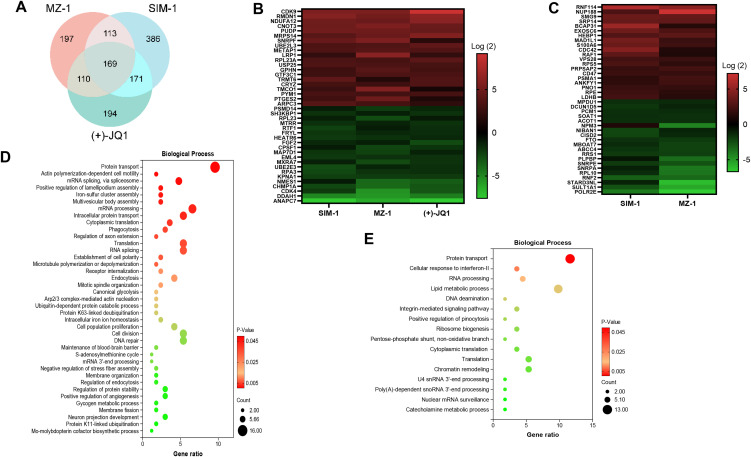
Global protein profile changed by selective BET inhibitors or degraders in KSHV-infected immortalized endothelial cells. **(A)** Protein profile changed by SIM-1, MZ-1 or (+)-JQ1 from TIVE-LTC was identified using proteomics/LC-MS analysis. The intersection analysis showed significantly altered proteins (FDR < 0.05) within TIVE-LTC treated by SIM-1, MZ-1 or (+)-JQ1, respectively. **(B-C)** Heat maps showed top 20 “common” proteins changed in SIM-1, MZ-1 or (+)-JQ1 treated cells, and top 20 “unique” proteins only changed in SIM-1 or MZ-1 treated cells. **(D-E)** The enrichment analysis of “common” proteins changed by SIM-1, MZ-1 or (+)-JQ1, and “unique” proteins only changed by SIM-1 or MZ-1, respectively.

We next analyzed global protein expression changes induced by selective BET inhibitors and degraders. Proteomic analysis identified 169 proteins significantly altered across MZ-1-, SIM-1-, and (+)-JQ1-treated TIVE-LTC cells, and 113 proteins uniquely altered in MZ-1- and SIM-1-treated cells (Fig 7A). Heat maps showed top 20 “common” proteins changed in MZ-1, SIM-1 or (+)-JQ1 treated cells, and top 20 “unique” proteins only changed in MZ-1 or SIM-1 treated cells, respectively (Fig 7B and 7C). The enrichment analysis of these “common” or “unique” proteins indicated that these two groups of proteins only had limited overlapped functional categories such as protein translation and transport, RNA processing, while the “unique” group had many its own functional categories such as cellular response to interferon, lipid metabolic processing, DNA deamination and ribosome biogenesis (Fig 7D and 7E). Future studies will focus on understanding why MZ-1, SIM-1 and (+)-JQ1 all affect BRD signaling, but resulting in different biological consequence (e.g., only MZ-1 or SIM-1 can inhibit the growth of KSHV-infected immortalized endothelial cells). Further work is also needed to define the roles of these unique protein candidates in MZ-1/SIM-1-mediated growth inhibition.

### MZ-1 and SIM-1 significantly increase viral lytic reactivation in low dose of Dox-induced iTIME.219 cells

**Fig 8:**
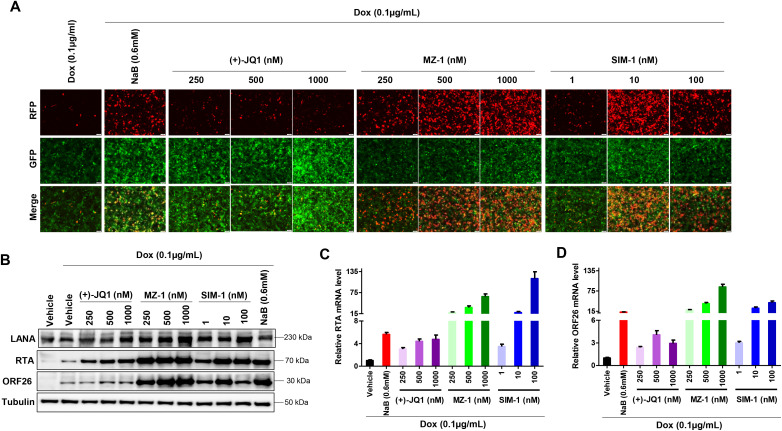
MZ-1 and SIM-1 significantly increase viral lytic reactivation in low dose of Dox-induced iTIME.219 cells. **(A)** The iTIME.219 cells were treated by indicated concentrations of compounds in conjunction with Dox (0.1 µg/mL), then GFP/RFP expression were evaluated at 72 h post-treatment as a measure of lytic reactivation. NaB (sodium butyrate) was used as a positive control. **(B-D)** Protein expression and gene transcription were measured by using Western blot and RT-qPCR, respectively. Error bars represent S.D. for 3 independent experiments.

We examined the impacts of MZ-1 or SIM-1 on viral gene expression in low dose of Dox-induced iTIME.219 cells. Our results showed that these PROTACs treatments significantly increased viral lytic reactivation from low dose of Dox-induced iTIME.219 cells when compared to Dox alone or Dox plus (+)-JQ1 groups (Fig 8A). Interestingly, instead of reducing LANA expression, MZ-1 and SIM-1 appeared to increase LANA levels under these conditions, suggesting that lytic cells may respond differently from latent cells. Our additional data confirmed that MZ-1 or SIM-1 treatment dramatically increased viral lytic genes such as RTA and ORF26 at both transcriptional and expressional levels, respectively (Fig 8B–8D).

## Discussion

In the current study, we compared the potency of various BET inhibitors and degraders in suppressing the growth of KSHV-infected immortalized endothelial cells. Notably, only MZ-1 and SIM-1— two BRD4-targeting PROTAC degraders — exhibited inhibitory effects. Consistent with these findings, only these two PROTACs (but not other BET inhibitors or degraders) effectively reduced LANA expression and induced degradation of BRD2 and BRD4 in KSHV-infected immortalized endothelial cells. It remains unclear why other BRD4 PROTACs tested, such as ARV-825 and dBET1, failed to induce target protein degradation in these cells. Notably, MZ-1 and SIM-1 are von Hippel–Lindau (VHL)-based PROTACs, whereas ARV-825 and dBET1 are cereblon (CRBN)-based PROTACs [28,29]. Since CRBN and VHL are the two most commonly exploited E3 ligases in PROTAC design, one possible explanation is that VHL is more highly expressed than CRBN in KSHV-infected endothelial cells, or that CRBN function is impaired in this context. However, these possibilities require further experimental validation.

Our data also showed that KSHV-infected immortalized endothelial cells exhibit strong resistance to all BET inhibitors tested, including (+)-JQ1. Proteomic analysis revealed that 169 proteins were significantly altered and shared among MZ-1-, SIM-1-, and (+)-JQ1-treated TIVE-LTC cells, whereas 113 proteins were uniquely altered in MZ-1- and SIM-1-treated cells. Enrichment analysis indicated that the shared (“common”) proteins were largely associated with BRD signaling and related cellular functions. In contrast, the uniquely altered proteins were enriched in pathways such as interferon response, lipid metabolism, DNA deamination, and ribosome biogenesis. These findings raise several important questions for future investigation: (1) why BRD4 PROTACs and BRD4 inhibitors differentially affect protein expression and function; (2) what roles these uniquely altered proteins play in MZ-1- or SIM-1-mediated growth inhibition; (3) how these proteins are mechanistically linked to BRD4 signaling; and (4) whether they contribute to cell survival and KSHV pathogenesis. For example, a recent study showed that KSHV manipulated ribosome biogenesis to produce specialized ribosomes that preferentially translate viral transcripts [30]. Specifically, increased association of ribosome biogenesis factors BUD23 and NOC4L, as well as the KSHV ORF11 protein, with small ribosomal subunit precursor complexes was observed during viral lytic replication [30]. Additionally, several uniquely altered proteins identified in our study have previously been implicated in KSHV infection and pathogenesis. For instance, CD47 is highly expressed on the surface of PEL cells, and anti-CD47 antibodies promote macrophage-mediated phagocytosis and inhibit ascites formation and organ invasion in a PEL xenograft model [31]. Similarly, the small GTPase CDC42 has been shown to play a role in early stages of KSHV infection [32,33].

We further demonstrate that targeting BRD2 and BRD4, either by PROTACs or RNAi, reduce LANA expression in KSHV-infected endothelial cells. Mechanistically, this reduction is not mediated by transcriptional repression, autophagy, or protein degradation, but rather by suppression of LANA translation through increased eIF2α phosphorylation. Supporting this mechanism, treatment with an eIF2α activator increased eIF2α phosphorylation and reduced LANA expression, whereas inhibition of eIF2α signaling restored LANA expression in MZ-1- or SIM-1-treated cells. These findings also explain why BRD PROTAC or RNAi treatments do not completely eliminate LANA protein, as they primarily block new protein synthesis rather than degrading existing protein pools. These results suggest that combining BRD PROTACs with recently developed LANA-targeting PROTACs [34] may provide a more effective strategy for eliminating LANA in infected cells. As a central regulator of the integrated stress response (ISR), eIF2α phosphorylation reduces global protein synthesis to conserve cellular resources under stress conditions. This process is mediated by four kinases— PERK, PKR, GCN2, and HRI— which inhibit translation initiation by modulating eIF2 recycling through eIF2B [35]. Each kinase is activated by distinct stress signals: PERK by endoplasmic reticulum stress, PKR by viral infection, GCN2 by amino acid deprivation, and HRI by heme deficiency or oxidative stress [35,36]. For example, Epstein-Barr virus (EBV) encoded LMP1 and human papillomavirus (HPV) encoded E7 protein can inhibit PERK activity in cancer cells via the interaction of the viral oncoproteins with PERK, leading to increased level of reactive oxygen species (ROS) and promoting tumor growth [37]. Another recent study reports that human cytomegalovirus (HCMV) pTRS1 protein can interact with protein phosphatase 1 (PP1) to suppress PKR/eIF2α signaling during virus infection [38]. Future work will focus on identification of which kinases are required for eIF2α phosphorylation from MZ-1 or SIM-1 treated KSHV-infected immortalized endothelial cells and exploring underlying molecular mechanisms.

In conclusion, our study identifies, for the first time, selective BRD4 PROTAC degraders, MZ-1 and SIM-1, as promising therapeutic candidates for KS. We also uncover a novel mechanism whereby targeting BRD2 or BRD4 suppresses LANA translation through induction of eIF2α phosphorylation. These findings may facilitate the development of more effective BET-targeted therapies for KSHV-related malignancies.

## Materials and methods

### Cell culture and reagents

HUVEC, TIME and KSHV long-term-infected immortalized endothelial cell lines, TIVE-LTC and iTIME.219 were cultured as described previously [39,40]. All experiments were carried out using cells harvested at low (< 20) passages. All of compounds used in this study were purchased from Selleck Chemicals or Sigma.

### Cytotoxicity assay

The cytotoxicity was measured using the WST-1 cell proliferation assays (Roche). Briefly, after the period of treatment of cells, 10 μL/well of cell proliferation reagent, WST-1 (4-[3-(4-Iodophenyl)-2-(4-nitro-phenyl)-2H-5-tetrazolio]-1,3-benzene disulfonate), was added and incubated for 3 h at 37°C in 5% CO_2_. The absorbance of samples was measured by using a microplate reader at 490 nm. Data was normalized as the inhibition relative to the vehicle control.

### RNA interference (RNAi)

For RNAi assays, On-Target plus SMARTpool small interfering RNA (siRNA; Dharmacon) targeting BRD2, BRD3, BRD4, c-Myc, or negative control siRNA were delivered using the DharmaFECT transfection reagent as recommended by the manufacturer.

### Western blot

Total cell lysates (20 μg) were resolved by 10% SDS–PAGE, transferred to nitrocellulose membranes, and immunoblotted with antibodies to LANA (Advanced Biotechnologies), RTA, ORF26 (Helmholtz-Munich), BRD2, BRD3, BRD4, c-Myc, phosphor-eIF2α/total-eIF2α, and Tubulin as a loading control (Cell Signaling). Immunoreactive bands were identified using an enhanced chemiluminescence reaction (Perkin-Elmer), and visualized by autoradiography.

### RT-qPCR

Total RNA was isolated by using the RNeasy Mini kit (Qiagen), and cDNA was synthesized using a SuperScript III First-Strand Synthesis SuperMix Kit (Invitrogen). Specific primers used for amplification of individual target gene were listed in S1 Table. The amplification was carried out using an iCycler IQ Real-Time PCR Detection System, and cycle threshold (Ct) values were tabulated in triplicate for each gene of interest in each experiment. “No template” (water) controls were used to ensure minimal background contamination. Using mean Ct values tabulated for each gene, and the paired Ct values for β-actin gene as a loading control, the fold changes for experimental groups relative to assigned control groups were calculated by using automated iQ5 2.0 software (Bio-rad).

### Polysome profiling assay

Ribosome-loaded RNA was isolated essentially as previously described [41]. Cells treated with SIM-1 and MZ-1 for 24 h, then treated with 100 µg/mL cycloheximide for 10 min. Cells were washed twice in PBS and lysed in ribosome lysis buffer (10 mM Tris-HCl pH 7.4, 5 mM MgCl_2_, 100 mM KCl, 1% Triton X, Protease inhibitor, 2 mM DTT, 100 mg/mL cycloheximide and RNase inhibitor). One tenth of the lysate was then used for RNA isolation for the “input” sample. The rest of the lysates were sheared by passage through a 26-gauge needle before clarification by centrifugation at 1,300 g for 10 min. The density gradient sucrose liquid was laid the night before. After that, the supernatant was covered on 10%-50% sucrose and then centrifuged at 164,000 g for 2 h, 4°C. RNAs from different ribosomal pellet layers were extracted using TRIzol. RNAs isolated from the input and ribosome loaded fractions then underwent RT-qPCR to quantify GAPDH and LANA mRNA levels.

### Proteomics analysis

TIVE-LTC were treated with SIM-1, MZ-1, (+)-JQ1 or vehicle for 24 h, then collected for proteomics analysis (5x10^6^ cells/sample with four replicates). Protein samples were reduced, alkylated, and purified by chloroform/methanol extraction prior to digestion with sequencing grade modified porcine trypsin (Promega). Tryptic peptides were then separated by reverse phase XSelect CSH C18 2.5 µm resin (Waters) on an in-line 150 x 0.075 mm column using an UltiMate 3000 RSLCnano system (Thermo). Peptides were eluted using a 90 min gradient from 98:2–65:35 buffer A:B ratio. Buffer A = 0.1% formic acid, 0.5% acetonitrile; Buffer B = 0.1% formic acid, 99.9% acetonitrile. Eluted peptides were ionized by electrospray (2.4 kV) followed by mass spectrometric analysis on an Orbitrap Eclipse Tribrid mass spectrometer (Thermo). MS data were acquired using the FTMS analyzer in profile mode at a resolution of 120,000 over a range of 375–1200 m/z. Following HCD activation, MS/MS data were acquired using the ion trap analyzer in centroid mode and normal mass range with a normalized collision energy of 30%. Proteins were identified by database search using MaxQuant (Max Planck Institute) with a parent ion tolerance of 3 ppm and a fragment ion tolerance of 0.5 Da. Scaffold Q + S (Proteome Software) was used to verify MS/MS based peptide and protein identifications. Protein identifications were accepted if they could be established with less than 1.0% false discovery and contained at least 2 identified peptides. Protein probabilities were assigned by the Protein Prophet algorithm [42]. The mass spectrometry proteomics data have been deposited to the ProteomeXchange Consortium via the PRIDE partner repository with the dataset identifier PXD075129.

### Fluorescence detection

The iTIME.219 cells latently carry a recombinant rKSHV.219 virus and a doxycycline (Dox)-inducible gene expression system for expression of viral replication and transcription activator (RTA) protein, of which expression is essential and sufficient for triggering KSHV reactivation [40]. The rKSHV.219 contains two fluorescent protein genes encoding the green fluorescent protein (GFP) and red fluorescent protein (RFP), which are derived from the EF-1α promoter and KSHV lytic PAN promoter, respectively [43]. The cells were treated by Dox (0.1 µg/mL) in combination with tested compounds at concentrations and time-points as indicated, then GFP and RFP expression were detected by a fluorescent microscopy (Olympus DP80).

### Statistical analysis

Significant differences between experimental and control groups were determined using the two-tailed Student’s *t*-test. The 50% Cytotoxicity Concentrations (CC_50_) were calculated from the dose-response curves using GraphPad Prism 10.

## Supporting information

S1 Table Primer sequences for RT-qPCR. (DOCX)

S1 Fig The potency of selective BET inhibitors or degraders in growth inhibition of KSHV-infected immortalized endothelial cells. (**A-B**) Cells were treated by different BET inhibitors or degraders for 48 h, then cell proliferation was measured by using the WST-1 assay. Error bars represent S.D. for 3 independent experiments.
(DOCX)

S2 Fig The impacts of MZ-1 and SIM-1 on target protein expression in *de novo* KSHV-infected HUVEC. HUVEC were infected by KSHV (MOI ~ 5) for 48 h, then treated by indicated concentrations of MZ-1 or SIM-1 for 24 h. Protein expression was measured by using Western blot.
(DOCX)

S3 Fig The impacts of selective BET inhibitors or degraders on target protein expression in KSHV-infected immortalized endothelial cells. (**A-B**) Cells were treated by indicated concentrations and time of SIM-1, MZ-1 or (+)-JQ1, then protein expression was measured by using Western blot.
(DOCX)

S4 Fig MG132 or VH 032 alone treatments do not affect LANA, BRD4 or c-Myc expression in KSHV-infected immortalized endothelial cells. (**A-B**) TIVE-LTC were treated by indicated concentrations of MG132 or VH 032 for 48 h, respectively, then protein expression was measured by using Western blot.
(DOCX)

S5 Fig Direct knockdown of c-Myc does not affect LANA and BRD4 expression in KSHV-infected immortalized endothelial cells. TIVE-LTC were transfected with Myc-siRNA or non-target control siRNA (si-NC) for 72 h, then protein expression was measured by using Western blot.
(DOCX)

S6 Fig MG132 or Chloroquine treatments cannot restore LANA expression in BRD2 or BRD4 knockdown cells. TIVE-LTC were pretreated with MG132 or Chloroquine, then transfected with BRD2-siRNA or BRD4-siRNA for 72 h. Protein expression was measured by using Western blot.
(DOCX)

S7 Fig Bafilomycin A1 or Chloroquine treatments cannot restore LANA expression in MZ-1 or SIM-1 treated TIVE-LTC cells. (**A-B**) Cells were treated by MZ-1 or SIM-1 with or without indicated concentrations of Bafilomycin A1 (A) or Chloroquine (B), respectively, then protein expression was measured by using Western blot.
(DOCX)

S8 Fig The impacts of MZ-1 and SIM-1 on eIF2α activity in TIME cells. Cells were treated by indicated concentrations of MZ-1 or SIM-1 for 24 h, then protein expression was measured by using Western blot.
(DOCX)
