# Supplementary material for: Targeting BRD2 and BRD4 inhibit the growth of KSHV-infected immortalized endothelial cells through suppression of LANA translation
Source: PLoS Pathog. 2026 Jun 1;22(6):e1014288. doi: 10.1371/journal.ppat.1014288 (PMC13235932; doi:10.1371/journal.ppat.1014288)
Supplement: S1 Table — (DOCX) [file ppat.1014288.s001.docx]

**Table S1. Primer sequences for RT-qPCR.**

| **Gene** | **Forward primers** | **Reverse primers** |
| --- | --- | --- |
| LANA | 5'-TCCCTCTACACTAAACCCAATA-3' | 5'-TTGCTAATCTCGTTGTCCC-3' |
| RTA | 5’-CACAAAAATGGCGCAAGATGA-3’ | 5’-TGGTAGAGTTGGGCCTTCAGTT-3’ |
| PF | 5’-CGAGTCTTCGCAAAAGGTTC-3’ | 5’-AAGGGACCAACTGGTGTGAG-3’ |
| ORF26 | 5’-GCTCGAATCCAACGGATTTG -3’ | 5’- AATAGCGTGCCCCAGTTGC-3’ |
| ORF17 | 5’-AGATTTTTCACGGGGGCTCTGG-3’ | 5’- TGGGCTGGACACTGGGTCTATTTC-3’ |
| GAPDH | 5’-GCTCCCTCTTTCTTTGCAGCAAT-3’ | 5’-TACCATGAGTCCTTCCACGATAC-3’ |
| β-actin | 5’-ATCGTGCGTGACATTAAGGAG-3’ | 5’-GGAAGGAAGGCTGGAAGAGT-3’ |
