# Supplementary figures and images for: Targeting BRD2 and BRD4 inhibit the growth of KSHV-infected immortalized endothelial cells through suppression of LANA translation
Source: PLoS Pathog. 2026 Jun 1;22(6):e1014288. doi: 10.1371/journal.ppat.1014288 (PMC13235932; doi:10.1371/journal.ppat.1014288)

**S1 Fig.**


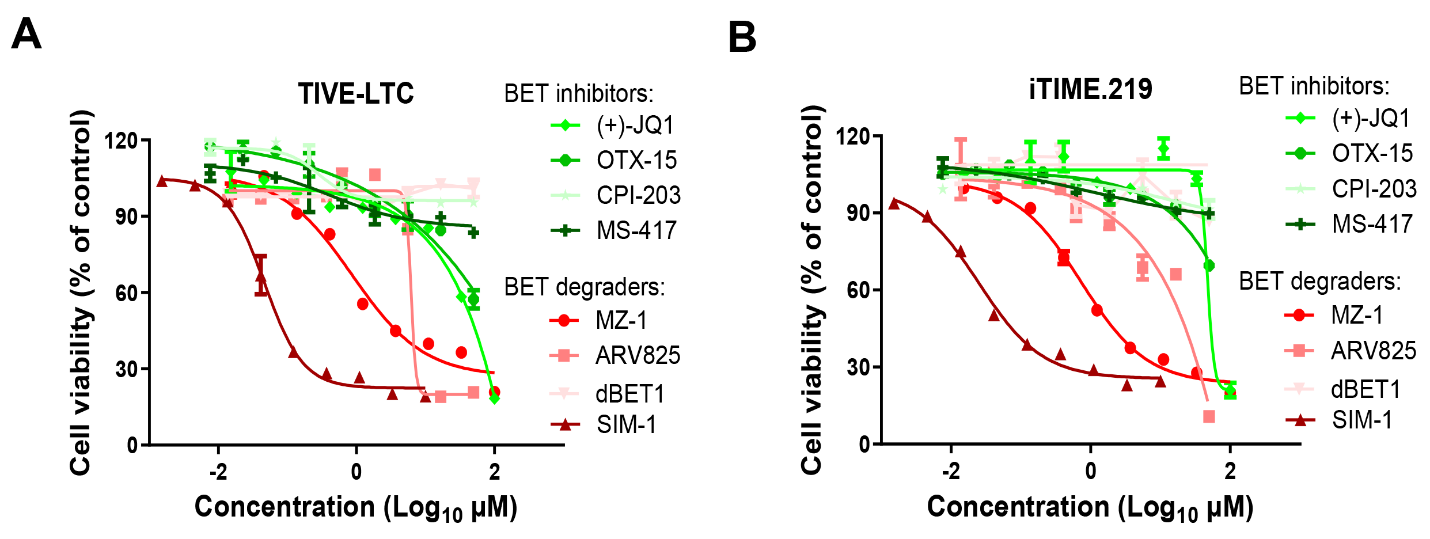

Supplement: S1 Fig — (A-B) Cells were treated by different BET inhibitors or degraders for 48 h, then cell proliferation was measured by using the WST-1 assay. Error bars represent S.D. for 3 independent experiments. (DOCX) [file ppat.1014288.s002.docx]

**S2 Fig.**

**
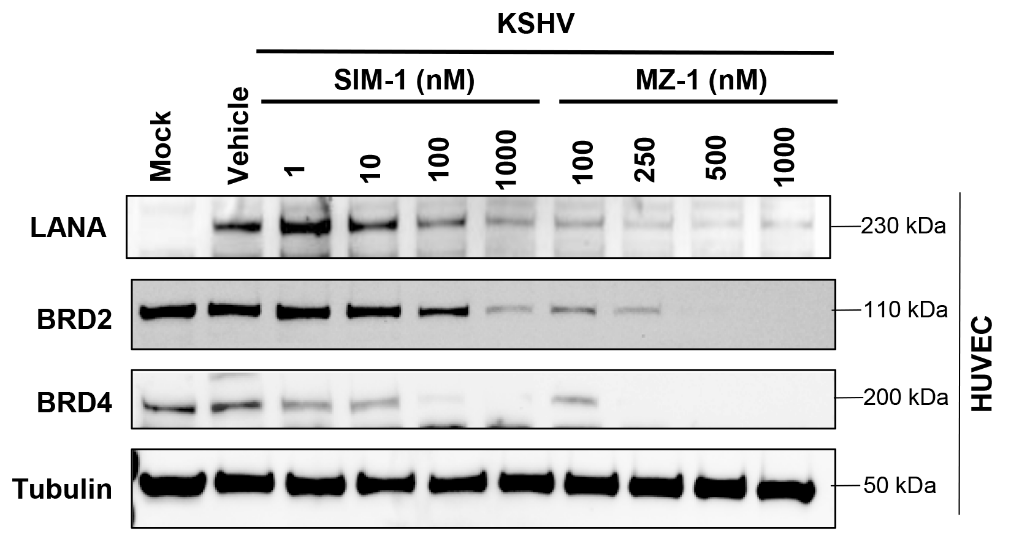
**

Supplement: S2 Fig — HUVEC were infected by KSHV (MOI ~ 5) for 48 h, then treated by indicated concentrations of MZ-1 or SIM-1 for 24 h. Protein expression was measured by using Western blot. (DOCX) [file ppat.1014288.s003.docx]

**S3 Fig.**

**
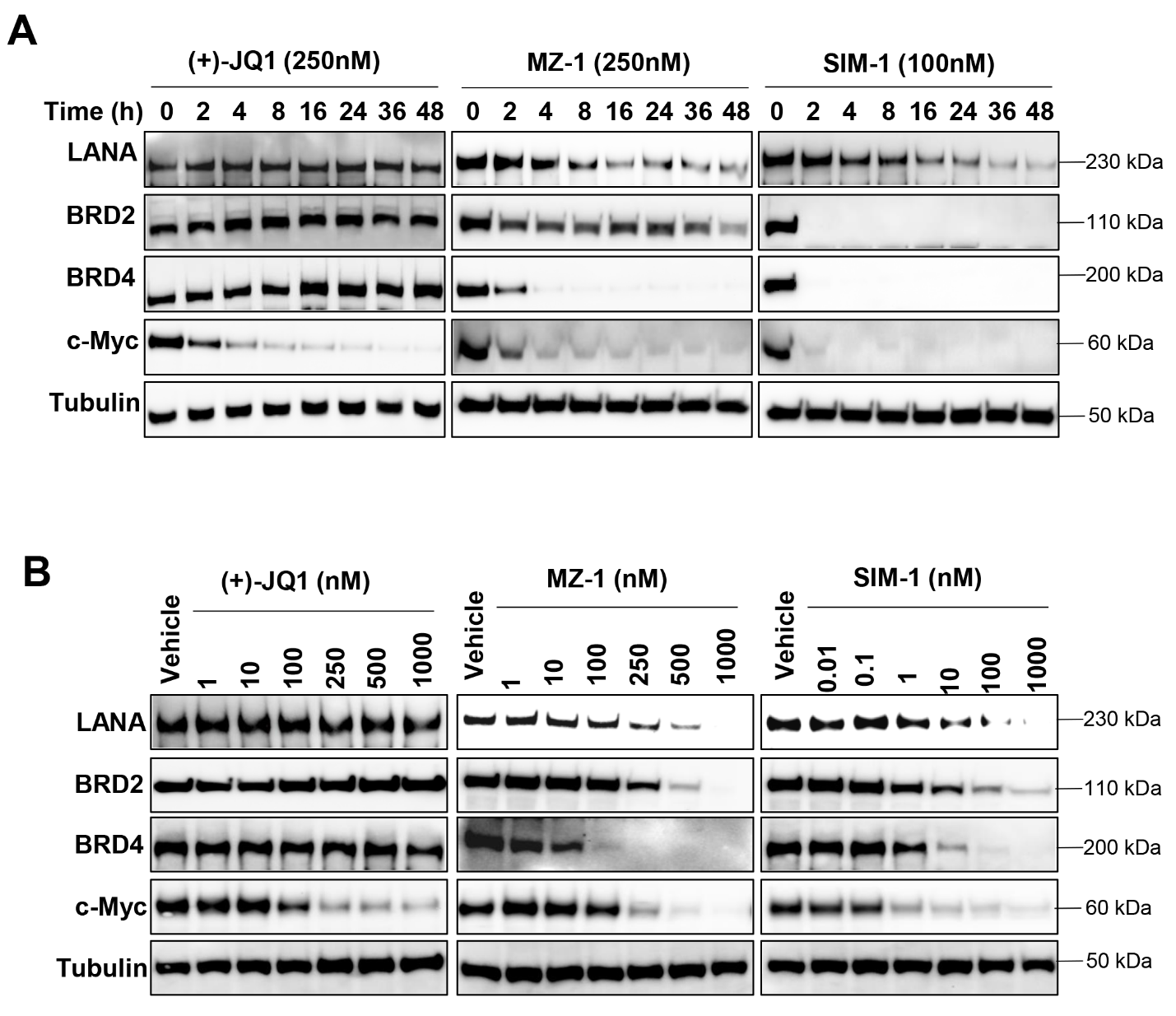
**

Supplement: S3 Fig — (A-B) Cells were treated by indicated concentrations and time of SIM-1, MZ-1 or (+)-JQ1, then protein expression was measured by using Western blot. (DOCX) [file ppat.1014288.s004.docx]

**S4 Fig.**

**
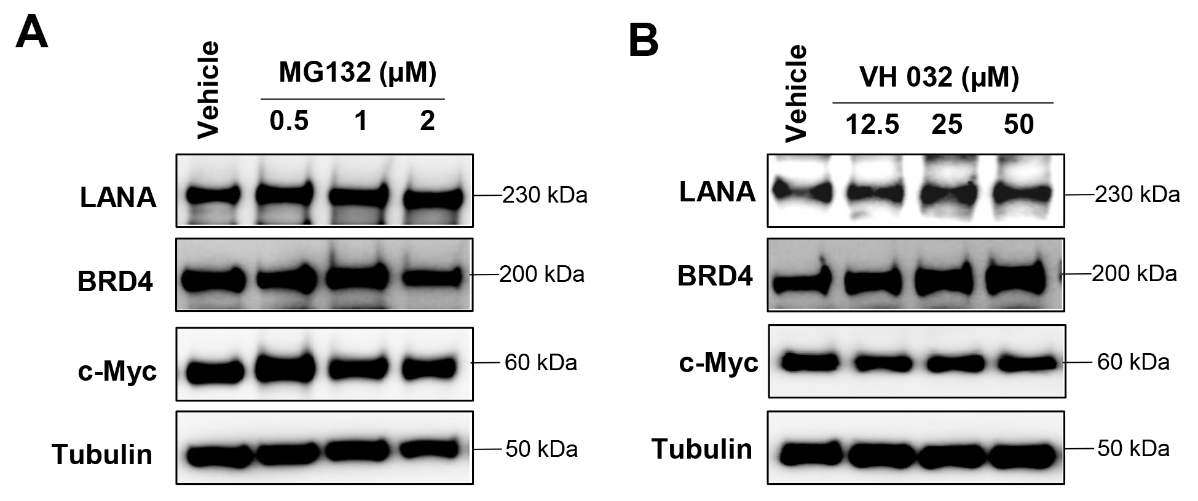
**

Supplement: S4 Fig — (A-B) TIVE-LTC were treated by indicated concentrations of MG132 or VH 032 for 48 h, respectively, then protein expression was measured by using Western blot. (DOCX) [file ppat.1014288.s005.docx]

**S5 Fig.**

**
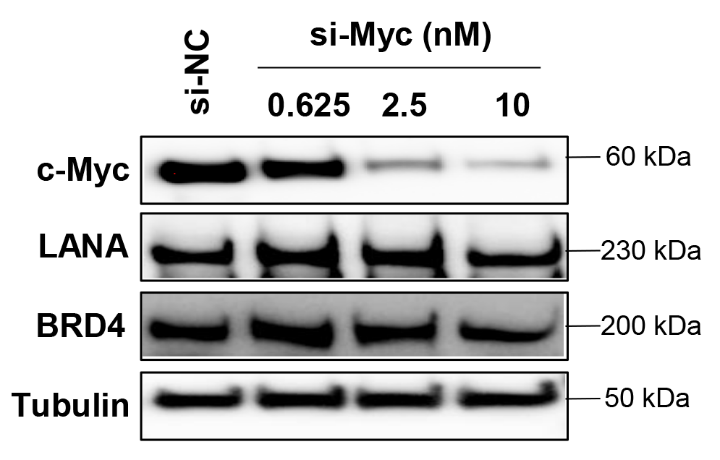
**

Supplement: S5 Fig — TIVE-LTC were transfected with Myc-siRNA or non-target control siRNA (si-NC) for 72 h, then protein expression was measured by using Western blot. (DOCX) [file ppat.1014288.s006.docx]

**S6 Fig.**

**
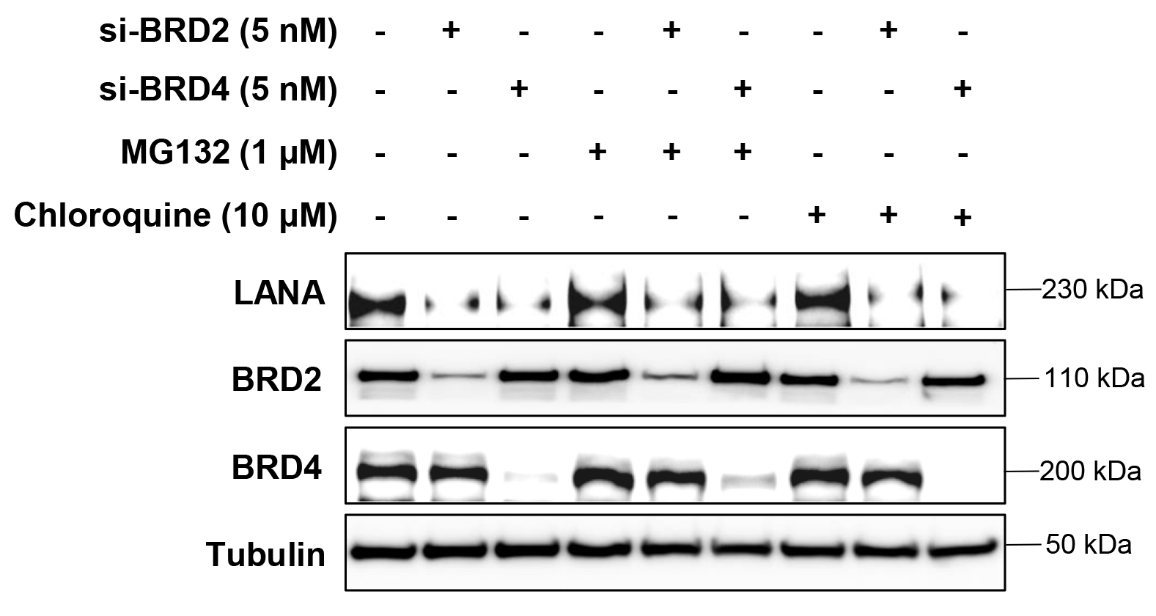
**

Supplement: S6 Fig — TIVE-LTC were pretreated with MG132 or Chloroquine, then transfected with BRD2-siRNA or BRD4-siRNA for 72 h. Protein expression was measured by using Western blot. (DOCX) [file ppat.1014288.s007.docx]

**S7 Fig.**


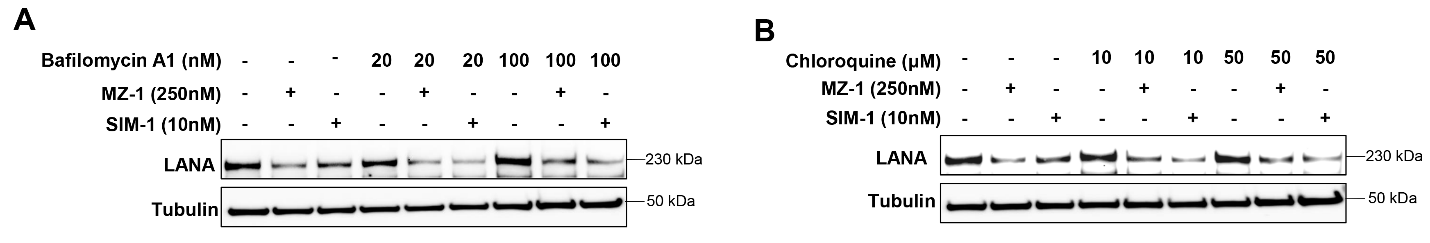

Supplement: S7 Fig — (A-B) Cells were treated by MZ-1 or SIM-1 with or without indicated concentrations of Bafilomycin A1 (A) or Chloroquine (B), respectively, then protein expression was measured by using Western blot. (DOCX) [file ppat.1014288.s008.docx]

**S8 Fig.**


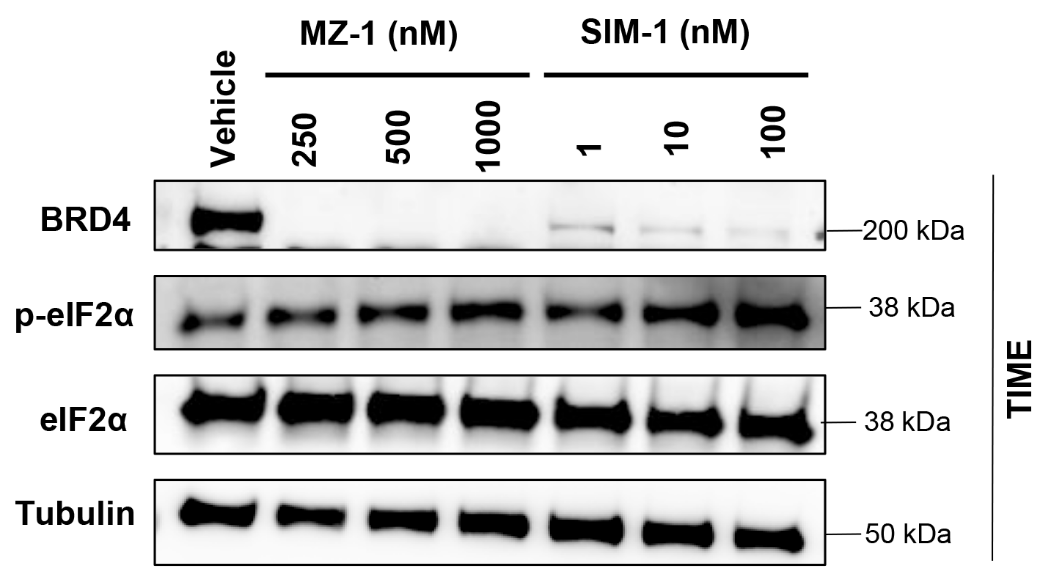

Supplement: S8 Fig — Cells were treated by indicated concentrations of MZ-1 or SIM-1 for 24 h, then protein expression was measured by using Western blot. (DOCX) [file ppat.1014288.s009.docx]
